# Economic Development as a Challenge for “De Facto States”: Post-Conflict Dynamics and Perspectives in South Ossetia

**DOI:** 10.1134/S2079970522700277

**Published:** 2022-12-20

**Authors:** A. B. Sebentsov, M. S. Karpenko, A. A. Gritsenko, N. L. Turov

**Affiliations:** Institute of Geography, Russian Academy of Sciences, Moscow, Russia

**Keywords:** “de facto states,” South Ossetia, unrecognized states, separatism, secession, state building, economic development

## Abstract

The significance of economic factors in the emergence and development of “de facto states” remains insufficiently studied and debatable. This article deals with the case of South Ossetia, one of the six unrecognized republics that emerged in the post-Soviet space. Based on the study of statistical data, secondary sources and expert interviews with representatives of local authorities, business, and the academic community, the authors analyzed structural changes and the state of the economy of South Ossetia and views on development prospects in the context of state building. It has been shown that as a result of large-scale assistance from Russia, a structurally weak hyperservice economy has formed, whose key industries depend on government demand and Russian investments. Limited economic self-sufficiency, cross-border settlement of Ossetians and Georgians, as well as numerous family ties contribute to the emergence of a variety of cross-border practices (shuttle trade, smuggling, and peculiar payment systems) that reduce social tensions. The case of South Ossetia confirms that unrecognized status is not in itself an obstacle to economic development, but the lack of external legitimacy limits access to markets and creates difficulties for financial and trade transactions. As a result, Eurasian integration has become rather a source of problems for this republic, creating obstacles difficult to overcome for local businesses in trade with Russia, the only EAEU country that recognizes the Republic of South Ossetia as a sovereign state. As a result, economic problems, along with security issues, serve as a key argument for South Ossetia’s support for the idea of joining the republic to Russia.

## INTRODUCTION

The events of recent decades have highlighted the acuteness of the crisis of statehood in many regions of the world and the process of fragmentation of the political space associated with it. Its concrete manifestation was the growth in the number of sovereign states. While in 1990 159 countries had full membership in the UN, by the beginning of the current decade there were 193. At the same time, along with recognized sovereign states, a large number of polities that do not have international recognition, but steadily control their own territory, have formed (Popov, 2015). At one pole of this heterogeneous group, there are at least 40 polities with extremely mobile borders, which are usually classified as uncontrolled territories (Sebentsov and Kolosov, 2012; Kolosov et al., 2021), at the other pole there are 13 “de facto states” with most of the hallmarks of a sovereign state, with the exception of broad international recognition (Zayats, 2020). There are six such entities in the post-Soviet space, two of which, Abkhazia and South Ossetia, belong to the category of partially recognized states.

The growing attention of researchers to uncontrolled territories in general and “de facto states” in particular is associated, first, with the growth of secessionist movements and territorial conflicts in different parts of the world, which have emphasized the task of finding the causes of contradictions that threaten the integrity of nation states, searching for opportunities and means to overcome them (Rabasa et al., 2007; Lamb, 2008). Second, of interest is the rivalry of world political actors involved in the process of conflict resolution, which led to the formation of new polities. Post-Soviet unrecognized and partially recognized states, for example, are often viewed in the context of geopolitical confrontation and the clash of interests between Russia and the West. A number of researchers (Lynch, 2002; Kupchan, 2005; Umland, 2014) have emphasized the decisive role of Russia in ensuring the security of the unrecognized republics and maintaining their political status quo.^1^

Third, there is a general turning of researchers to the study of internal factors of the consolidation of “de facto states” and the processes of (pseudo)state building (Il’in et al., 2010). The political systems and institutions of the unrecognized states, their socioeconomic situation, the development of certain sectors of the economy (Golunov and Zotova, 2021), public opinion, and the processes of formation of civic identity (O’Loughlin and Kolosov, 2017) were analyzed in increasing detail. Comparative studies have become widespread (Popescu et al, 2006; Broers et al., 2015; Markedonov, 2018; Zayats, 2020).

The Republic of South Ossetia has also been an object of study more than once. For example, P. Kolstø and H. Blakkisrud, comparing the formation of the statehood of South Ossetia, Abkhazia, and Nagorno-Karabakh in the conditions of a military conflict (Kolstø and Blakkisrud, 2008) and the post-conflict period (Blakkisrud and Kolstø, 2012), noted that South Ossetia suffers from a lack of economic resources to create full-fledged state institutions and is therefore doomed to constantly follow the vector of Russian politics. B. Baarová (2019) also emphasized the special role of Russia in solving the socioeconomic problems of the republic, which gives her reason to talk about “strange independence” that is paid for mainly with Russian money. T. Hoch (2020, p. 68, 70) writes about the “puzzling reality” in which the South Ossetian society, on the one hand, “seeks independence, but on the other, it constantly seeking to join Russia.” The author considers the security problems of the republic and the idea of reunification of the divided nation to be the main arguments for joining Russia. Representative polls conducted by a group of Russian and American geographers in South Ossetia in November 2010 showed that 81% of the South Ossetian population wants to become part of Russia (O’Loughlin et al., 2014).

The purpose of this paper was to analyze the role of the economy in the formation of the statehood of the Republic of South Ossetia on the basis of modern theoretical ideas about the evolution of the statehood of unrecognized and partially recognized states, developed by N. Caspersen.

The tasks of the paper include:

— periodization of the co-evolution of statehood and the economy of the Republic of South Ossetia in accordance with the theoretical scheme of Caspersen;

— assessment of modern features and problems of the development of the economy of the Republic of South Ossetia, especially during the COVID-19 pandemic;

— analysis of the relationship between ideas about possible pathways of economic development in political circles, media space, the expert community and the prospects for real sovereignty of the Republic of South Ossetia.

In the first part of the article, we will briefly characterize the theoretical approaches used in the study of the evolution of the statehood of unrecognized and partially recognized states, as well as the data collected and the methods used in this paper. We will then characterize the evolution of the economy at different stages of the state development of South Ossetia. In the last part, we will pay special attention to how the problems and opportunities for economic development are seen in expert and socio-political discourse. All this will allow not only assessing the current state of the economy of South Ossetia, but also approaching the question of what role the economy plays in understanding the prospects of their own statehood by the South Ossetian society.

## THE THEORETICAL FRAMEWORK OF THE RESEARCH

Most of the modern research connects the evolution of unrecognized states with the development of internal and external sovereignty, and hence the formation of state capacity. Thus, N. Caspersen (2012) noted that the key role for successful state building is played by internal sovereignty and related “internal resources for state building,” without which international recognition has symbolic results. External sovereignty opens up access to the system of international relations and sources of external support. A similar opinion was shared by D. Lynch (2002), who believed that it is critically important for an unrecognized state to ensure, first of all, the essence of state viability. Caspersen,^2^ characterizing the process of achieving state stateness, suggested that it can be divided into several often overlapping phases (Caspersen, 2012)

*The first phase* is associated with the establishment of power control over the territory of an unrecognized state. It constitutes the fundamental basis of state building, without which the whole project of statehood is in jeopardy. Another important task of this period is to establish control over own borders. The issue of ensuring military security has a natural priority.

*The second phase* is associated with the assertion of a monopoly on “legitimate” violence. At the beginning of this stage, the integration of irregular paramilitary groups, challenging the civilian government, into the regular armed forces usually occurs. The future of state building largely depends on the success of this process, since most often personal interests of authoritative field commanders run counter to the task of building effective state institutions. Another major task of this period is the formation of authorities, administrative structures, institutions, and procedures for the reproduction and distribution of public goods.

An active post-war recovery usually ends the second and opens *the third phase* of development, in which questions of internal legitimacy become increasingly important. The main task of the government is to expand the range of public services provided. The issues of arranging the communal infrastructure are being resolved, the systems of healthcare, education and culture are being developed, which are assigned a special role in creating and maintaining a common identity.

According to Caspersen, the most important motive for the gradual evolution and transition of an unrecognized state from one phase to another is the issue of security. The inability to ensure the safety of the population of an unrecognized state within the framework of a mother state is often called the starting point of the conflict and the main justification for the need for secession. In the future, the constancy of the external threat, the very state of “nonrecognition” also generates the need for strong institutions, pushing unrecognized states towards de facto statehood and imposing on them an interest in institutional building (Caspersen, 2012). External pressure from regional and world powers, as well as the support of patron states, dictate certain standards of “external legitimacy” to unrecognized states, which allows civilian leaders to defeat field commanders by building state structures and establishing control over economic resources. However, external pressure in the context of the weakness of the internal institutions of the state sometimes borders on interference in internal affairs and can also be considered as a threat to security.

We assume that the issue of economic stateness plays a central role here. On the one hand, patron states act as key drivers of the economy of unrecognized states, becoming intermediaries in their international transactions (financial transfers, trade in goods and services), providing access to their own markets (labor, goods, services and capital), rendering direct economic assistance. This dependence is greater the more isolated an unrecognized state turns out to be. On the other hand, the high participation of a patron state in the economy of an unrecognized state gives rise to economic and political dependence, which is painfully perceived by the elites and the population, creates a crisis of “sovereignty,” and contributes to the development of irredentist sentiments.

This suggests that the development of an unrecognized state and its economy can largely be characterized and understood through the prism of securitization theory, which interprets security not as an objective state of affairs, but as a political practice reflected in discourse (Buzan et al., 1998). This discourse is aimed at changing the priorities of social development and transferring politics from a procedural plane to an emergency one. Securitization as an extreme form of politicization postulates the presence of an existential threat to some object that has value in the eyes of the audience. At the same time, emergency and urgent measures are proposed to eliminate the threat, requiring a retreat or abandonment of established procedures and institutions. Thus, everything that calls into question the internal and external sovereignty, as well as the viability of an unrecognized state, is perceived as an “existential threat” and is subject to securitization (Kolstø and Blakkisrud, 2010). As a result of securitization, new (pseudo)state institutions and norms appear, old procedures are corrected, and, with favorable developments, there is an increase in stateness.

## MATERIALS AND METHODS

The study relies on three main groups of information sources.

The basis of the first group of sources was official statistics and departmental data, which were used to characterize the current and historical socioeconomic situation of the Republic of South Ossetia. Most of the statistical data used by the authors is available in the form of separate tables and collections on the website of the State Statistics Office of the Republic of South Ossetia,^3^ a small part of the materials (regarding individual enterprises, budget, foreign trade) were obtained through an official request, as well as during an expedition to the Republic of South Ossetia in October 2020. To compare the socioeconomic situation of the Republic of South Ossetia with its neighbors, materials from Rosstat, the National Statistical Service of Georgia and the World Bank were used.

The second group of data was the materials of the Res State News Agency, placed in the public domain on the Internet. Res is the largest media outlet in South Ossetia, which publishes official reports of state authorities of the republic, the most important articles from other republican media and produces its own content. For the analysis, 2010 (the boundary of the second and third phases of state building) and 2019 (the third phase) were taken. Initially, 9010 messages were selected: 3240 for 2010 and 5770 for 2019; however, due to numerous repetitions, 2028 and 2545 messages were taken for further analysis, respectively. These materials were used for discourse analysis, whose purpose was to study the dominant ideas in the media space and political circles about the current state and prospects for the development of the South Ossetian economy and its own stateness. An additional source of information was the Strategy for the Socioeconomic Development of the Republic of South Ossetia until 2030 (hereinafter referred to as the Strategy).^4^ It helped clarify some of the discussions that took place during its development in 2010 and the debate about its possible revision in 2019.

The third group of sources was 26 semi-structured interviews conducted in the Republic of South Ossetia by the authors of the article in October 2020. Most of the interviews were conducted with officials (11), that is, representatives of the Presidential Administration (3), the Ministry of Foreign Affairs (1), the Ministry of Culture (1), the Ministry of Economic Development (2), the Administration of the Leningor district (2), Embassy of Russia in South Ossetia (1), Federal Customs Service of Russia in South Ossetia (1). The second group of informants consisted of representatives of the scientific community (9) from the South Ossetian State University (8) and the South Ossetian Research Institute (1). The third group of informants consisted of business representatives (3): heads of the Chamber of Commerce and Industry (2) and one of the top managers of the Ironsan winery (1). The smallest group of informants was representatives of the media (1) and civil activists (2), including one representative of pro-Georgian activists (the interview was conducted in Leningor). The interview guide contained both general questions regarding the assessment of the socioeconomic situation of the Republic of South Ossetia, the nature of relations with Russia and its neighboring regions, Georgia and other foreign states, as well as specific questions that changed depending on the competence of the informant. Interviews with experts contributed to a better interpretation of the data obtained from the sources listed above, and made it possible to obtain information about the existing cross-border practices of the local population and residents of neighboring countries.

## THE EVOLUTION OF ECONOMIC DEVELOPMENT: FROM THE SOVIET PAST TO (IN)DEPENDENCE

As part of the Georgian SSR, the South Ossetian Autonomous Oblast (SOAO) was the least developed region with an industrial-agrarian economy. By 1989, 2% of the population of the union republic (95.8 thousand people) lived in it, and the contribution to the economy was only 1.1% of its GDP, 0.8% of industrial and 0.7% of agricultural production. The largest enterprises (Elektrovibromashina and Emalprovod, as well as mechanical and woodworking plants) participated in the all-union division of labor, but most of the sales were carried out through Georgia. The agro-industrial complex played an important role: the production of wines, beer, canned food, etc. (Champain et al., 2004; Zayats, 2004).

In the 1980s, in the context of the rise of the national movement in all the union republics, the relative economic backwardness, interpreted as part of the policy of discrimination by the authorities of the Georgian SSR, was one of the key arguments in favor of the region’s sovereignty. By 1989, mutual claims were increasingly moving to the plane of interethnic relations and contributed to the transition of the conflict into a hot phase.

Using Caspersen’s scheme, three successive phases can be distinguished in the development of the statehood and economy of the Republic of South Ossetia.

*The first phase* of state building covers the period from 1989 to 1994. It was during this period that the “hot phase” of the conflict occurred, officially ending on June 24, 1992 with the signing of the Sochi agreements, according to which the parties pledged to cease fire and withdraw their armed formations from the zone of contact. According to official materials of those years, hostilities led to the loss of a significant part of the housing stock, the destruction of the most significant transport infrastructure (including the Tbilisi-Tskhinvali railway), as well as the shutdown of almost all industrial enterprises in the region.^5^

The flow of refugees from South Ossetia and adjacent regions of Georgia resulting from the conflict was estimated at 130 000 people,^6^ while the population has more than halved. The key event of this phase of development was the referendum held on January 19, 1992, in which the vast majority of the local population voted for the independence of the Republic of South Ossetia and its subsequent reunification with Russia. However, by the end of the first phase, it was not possible to establish full control over the contested territory. Until August 2008, most of the Leningor district remained under the control of Georgia or pro-Georgian forces, while a significant part of the Znauri and Tskhinvali districts resembled a “patchwork of Georgian and Ossetian villages with polar political allegiance” (Blakkisrud and Kolstø, 2012). Humanitarian aid from Russia, along with subsistence farming, was the most important source of economic benefits for the population and the emerging South Ossetian statehood.

The beginning of *the second phase* of the development of statehood, apparently, occurred in 1993–1995. According to A. Markin,^7^ during this period, a kind of “military democracy regime was formed in the Republic of South Ossetia, in which the decisive role was played by field commanders who distinguished themselves at the stage of armed struggle and had significant political and economic ambitions.” The absence of clear advantages for one of the clans and Russia’s policy of maintaining the status quo contributed to the formation of political pluralism and relatively competitive elections. The need for internal and external legitimation spurred local elites to active institutional building (the formation of executive and legislative bodies, the introduction of the institution of presidency, etc.) and the relative centralization of power. Initially, active institutional building did not allow any noticeable change in the socioeconomic situation of the population. Its main sources of livelihood were personal subsidiary farms, work in state authorities, and mediation in semi-legal trade in a wide range of goods on the borders with Georgia and Russia.

The basis of the economy of the emerging South Ossetian state was the Ergneti market located in the suburbs of Tskhinvali, a symbol of Russian–Georgian informal cross-border trade (Champain et al., 2004; Gotsiridze, 2004). In the second half of the 1990s–early 2000s, the Ergneti market was one of the largest in the South Caucasus, and South Ossetia itself was a kind of free economic zone. Fuel, manufactured goods, vodka, flour were brought from Russia to South Ossetia, and citrus fruits, apples, grapes, herbs, wines, alcohol, and cigarettes were brought from Georgia. On the one hand, many Russian and Western observers noted that the Ergneti market performed a powerful “peacekeeping” function, facilitating unofficial daily contacts between business, South Ossetian and Georgian authorities, the local population, etc.^8^ On the other hand, in the same market, stolen cars were sold and arms and people were traded. Georgia’s attempt to regain control over the flow of goods across the border resulted in its conflict with South Ossetia in 2004. The loss of income from cross-border trade, in turn, reduced the influence of a number of authoritative field commanders (D. Tadeev, D. Sanakoev, D. Karkusov, etc.), contributed to the centralization of power in the hands of President E. Kokoity, and the growth of Russia’s influence.^9^ Thanks to the passportization of the local population, carried out by Russia in 2001–2004, Russian pensions and social benefits have become a significant source of income for the local population.

In *the third phase*, which began in 2008 after the hostilities involving Russia and its recognition of the independence of the republic, Russian assistance to South Ossetia became systemic and large-scale. Thus, from 2008 to 2011, within the framework of the Comprehensive Plan for the Restoration of the Republic of South Ossetia, Russia allocated over RUB 12 bln for the restoration of the most significant housing and communal services, social and transport infrastructure. Another RUB 7 bln was allocated from federal funds to cover the deficit of the state budget of the Republic of South Ossetia. Gazprom PJSC spent about RUB 10 bln on the construction of the Dzuarikau–Tskhinvali gas pipeline. At the expense of Federal Grid Company PJSC, the main power grids were modernized (Baarova, 2019). Since 2011, jointly developed and constantly updated investment programs to promote the socioeconomic development of the Republic of South Ossetia have become the main form of assistance.

By 2013–2014, these programs and the investment resources of Russian companies made it possible to solve the most acute problems of post-war reconstruction and determined the current socioeconomic situation in the republic.

## THE CURRENT STATE OF THE ECONOMY

International isolation, rupture of cross-border communications with the mother state, economic and transport blockade significantly complicate the functioning of the economy and foreign trade of unrecognized states, and limit the possibilities of their socio-economic development. With the exception of the Republic of China (Taiwan), none of the unrecognized and partially recognized states that exist today have been able to achieve significant success in economic development. However, even in this case, the help of the patron state played a decisive role (Sebentsov and Kolosov, 2012).

Russian economic assistance contributed to a noticeable growth in the economy of the republic: in 2015–2019, the GDP of South Ossetia increased by almost 40%. However, in terms of GDP per capita (in terms of PPP) from the neighboring states, the Republic of South Ossetia is comparable only to Abkhazia (whose GDP is only 20% higher), but is much inferior to the indicators of neighboring Russia (by 4–5 times) and Georgia (by 2–3 times). Differences are also great in comparison with the regions of neighboring countries. Thus, the GRP per capita of the nearest North Caucasian regions of Russia, which are also distinguished by the structural weakness of the economy, is 1.5–2 times greater than the GDP of South Ossetia (1.9 times in North Ossetia). Most of the Georgian regions are ahead of the Republic of South Ossetia in this indicator by an average of 2 times (the metropolitan Mtskheta-Mtianeti region, by 3 times).

Structurally, the economy of the Republic of South Ossetia remains unbalanced and weak. In 2019, according to official statistics, the service sector accounted for almost 81% of the generated gross value added, while about 35% of GDP was formed in the public administration sector, and another third was associated with the provision of social services: education (19.9%), healthcare (9.5%), etc. At least 65% of the employed population worked in all the listed branches of the public sector of the service industry. Transport and communications accounted for another 3.9% of the republic’s GDP. A significant role in the economy is played by construction (7.8% of GDP), which is carried out advantageously within the framework of the Russian investment program: it is in this sector that the highest average salary occurs, more than RUB 28 thous. (average for the country, RUB 19.6 thous.).

Despite the fact that the last decade in the economy of South Ossetia passed under the banner of reindustrialization, industry in 2019 accounted for only 7.3% of GDP. Initially, the greatest efforts were directed at the restoration and modernization of the leading enterprises of the Soviet era, such as the Bagiat bottling plant, the leader in the production of mineral water and one of the few profitable enterprises in the republic. A part of the production base of Emalprovod and Elektrovibromashina was also restored, but almost from the very beginning their activity turned out to be unprofitable; it was not possible to establish production relations both for marketing and for the supply of raw materials. Enterprises mainly produced noncore products (metal structures, corrugated board, etc.), provided industrial services, but more often and more profitably leased vacant premises.

The food industry has become the main direction of private and private-state investments. Russian investors have built the Ironsan winery, which produces wines and cognacs from local raw materials and wine materials purchased in Europe and Russia. In addition, the Naturplant bottling plant was put into operation, producing Edis premium mineral water, which is planned to be exported. The recently built Rastdon meat processing plant is under conservation, its owners are faced with a shortage of raw materials and the difficulty of marketing products to Russia. Large-scale restoration work contributed to the development of local production of building materials (the Construction Products Plant, Irbasalt). 

The most successful reindustrialization project was the restoration of clothing production at the Tskhinvali warp knitting factory (now BTK 4 Garment Factory—subsidiary of Russian BTK Group) in 2013. This enterprise produces various types of finished garments: military, construction, and since 2020, various personal protective equipment against infection. The launch of this relatively small production had a dramatic effect on the sectoral structure of the industry (Table 2). Thus, while in 2013, about 60% of industrial production was in the food industry, by 2017, the share of light industry had grown to 73.5%. The structure of South Ossetian exports also changed. While in 2010 it was based on mechanical products and metals (70.2%), as well as agricultural products (21.8%), in recent years about 90% is for light industry products, that is, BTK 4.

**Table 1.** Russian financial assistance to the budget of the Republic of South Ossetia in 2010–2020

Indicator	2010	2011	2012	2013	2014	2015	2016	2017	2018	2019	2020
Amount of subsidy, RUB bln	7.2	6.3	5.5	4.3	5.7	6.1	8.2	6.1	5.9	7.5	6.3
Share of subsidies in the revenue, %	98.7	93.5	84.2	89.9	91.8	88.3	90.1	84.7	78.6	84.2	82.1

*Compiled* by the authors according to the data of (Baarová, 2019), with clarifications according to the data of (Stat. Yearbook for 2020. Official Ed., State Statistics Office of the Republic of South Ossetia, Tskhinvali: IP O.S. Ikaev, 2021).

**Table 2.** The sectoral structure of industry in the Republic of South Ossetia, %

Sector	2013	2014	2015	2016	2017	2019
Machinery industry and metalworking	13.0	6.5	3.2	2.0	0.4	0.2
Forestry and woodworking industry	4.1	3.2	1.8	1.1	0.8	0.6
Flour and feed industry and baking industry	46.9	26.3	18.1	18.2	11.2	11.0
Other sectors of food industry	16.6	11.3	7.6	6.7	8.6	10.0
Production of building materials	4.0	2.1	1.8	5.0	3.4	4.0
Printing industry	9.4	7.0	3.4	3.4	2.2	4.0
Light industry	5.9	43.6	64.1	63.6	73.5	73.0

*Compiled* by the authors according to the data of (Stat. Yearbook for 2015. Official Ed., State Statistics Office of the Republic of South Ossetia, Tskhinvali: SSO RSO, 2016; Stat. Yearbook for 2020. Official Ed., State Statistics Office of the Republic of South Ossetia, Tskhinvali: IP O.S. Ikaev, 2021).

The situation is more complicated in agriculture, which accounts for no more than 0.4% of GDP. The volume of agricultural production after rapid recovery growth since 2017 has been steadily declining, even without inflation (in 2020, 81% compared to 2017). The structure of production is traditionally dominated by animal husbandry (about 80%). Almost all livestock and poultry are kept in personal subsidiary farms (over 93%), which are ready to produce a limited amount of products for sale. Despite numerous farming support programs, the number of farms has been steadily declining in recent years. Attempts to attract investment in horticulture have not brought tangible results either. The largest project, Gardens of Iryston (growing apples), also failed. A similar situation develops in viticulture. According to authorities, the main obstacle to the development of agriculture is the difficulty of marketing products to the food enterprises of the republic and to Russia. Representatives of the food industry see the problem in the fact that farmers cannot provide the required level of quality and consistency in the volume of products.

Limited economic self-sufficiency, the contact nature of the Russian–South Ossetian border, the cross-border settlement of Ossetians, as well as numerous family ties contribute to the emergence of various *cross-border practices* that reduce the social tensions and economic “isolation” of the Republic of South Ossetia. Neighboring regions of Russia provide opportunities to meet the needs in a wide range of goods and services (medical, educational, etc.), earn money and build a career, etc. The procedure for crossing the border is largely formal for residents of the Republic of South Ossetia and usually does not take more than 30–40 min, for residents of Russian regions, this is 60 min. Passage through the only checkpoint at Nizhny Zaramag is carried out mainly by private vehicles, less often by the Tskhinvali–Vladikavkaz bus.

The high cross-border mobility of the inhabitants of South Ossetia has become the reason for the emergence of the phenomenon of living “in two houses.” Many residents of North Ossetia, who are former refugees from South Ossetia, have second homes in Tskhinvali. Usually they are used as summer cottages or for rent to the Russian military and customs officers. Due to the high demand, the cost of renting apartments and houses is comparable to prices in the regional capitals of Central Russia. In the case of the departure of young people to work in Russia, elderly parents remain in the “second homes.” In addition, dual citizenship with Russia allows local residents to receive various kinds of Russian benefits and social benefits. In the Leningor district, where ethnic Georgians live compactly, similar cross-border practices are associated with neighboring regions of Georgia.

The COVID-19 pandemic has had a strong impact on the South Ossetian economy. In April 2020, the South Ossetian authorities closed the borders with Russia, which led to a shortage of everyday goods and a significant increase in prices. Subsequently, the governments of the two countries approved a list of entrepreneurs who were allowed to carry out cross-border transportation. The inability to travel to Russia for shopping and services led to a four-fold increase in wholesale and retail trade turnover in 2020, while the growth in gross value added of domestic trade made it possible to compensate for the decline in other industries (the share of trade increased from 2.3 to 14.3% of GDP).

In the context of the coronavirus pandemic, a two-fold drop in gross value added in industrial production and construction (by 3.5 and 2.3%, respectively), Russian assistance was especially important for maintaining social stability. In 2019–2020, it accounted for 81% of the revenue side of the budget of the Republic of South Ossetia (RUB 6.3 out of 7.6 bln) and 104.7% of the country’s GDP. About RUB 4.5 bln more was allocated to the Republic of South Ossetia for 2020–2022 within the framework of the Investment Program for the Promotion of the Social and Economic Development of the Republic of South Ossetia.

Thus, the economy of the Republic of South Ossetia is characterized by total dependence on Russia, its direct (subsidies, investments, etc.) and indirect (access to markets, financial and trade mediation) assistance. Cross-border practices of the local population are also associated with Russia, which compensate for the small capacity and narrowness of local markets (labor, goods and services, etc.) and have a stabilizing effect on the socioeconomic situation of citizens. It is not surprising that the problem of economic viability is one of the key issues of socio-political and expert discussion within the Republic of South Ossetia itself. 

## PATHWAYS OF ACQUISITION OF ECONOMIC STATENESS IN PUBLIC REPRESENTATIONS

As the statehood of the Republic of South Ossetia evolves and moves away from the pivotal events of 2008, the discourse of Res State News Agency is noticeably changing: this is manifested both in the rhetoric used and in the topics of the articles (see Fig. 2).

**Fig. 1.**
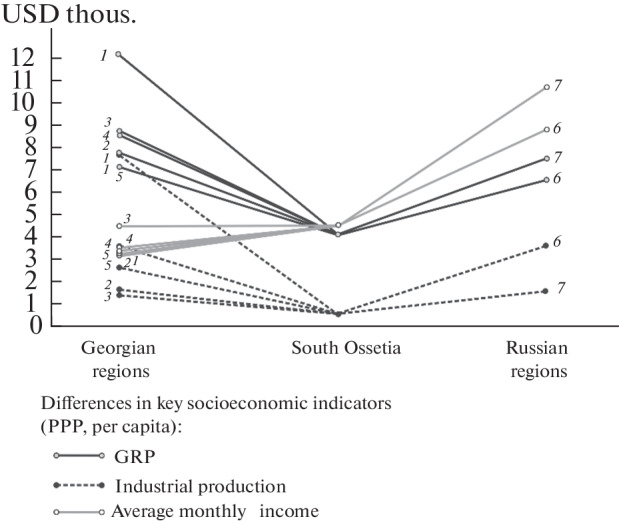
The socioeconomic gradients between South Ossetia and neighboring regions of Russia and Georgia, USD thous. in terms of PPP. *Compiled by* the authors according to: National Statics Office of Georgia. https://www.geostat.ge/en/modules/categories/93/regional-statistics; Stat. Yearbook for 2020. Official Ed., State Statistics Office of the Republic of South Ossetia, Tskhinvali: IP O.S. Ikaev, 2021; World Bank Open Data. https://data.worldbank.org/?lang=EN. Regions of Georgia: *1*, Mtskheta-Mtianeti; *2*, Samegrelo-Upper Svaneti; *3*, Racha-Lechkhumi and Lower Svaneti; *4*, Imereti; *5*, Shida Kartli. Regions of Russia: *6*, Karachay-Cherkess Republic; *7*, Republic of North Ossetia-Alania.

**Fig. 2.**
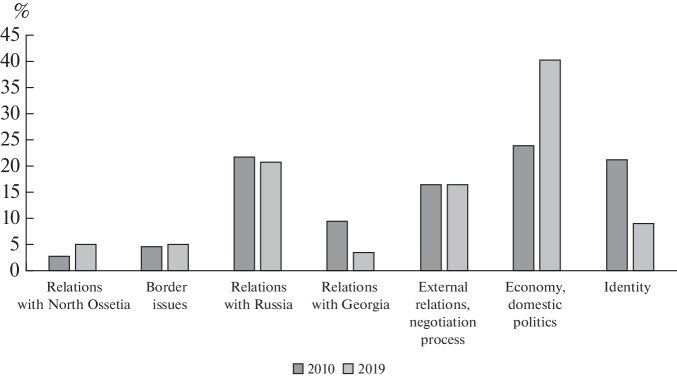
Main plots of the media discourse on sociopolitical topics in 2010 and 2019, % of the total number of articles.

About half of all articles in 2010 were related to “hard security” issues in one way or another. This topic is widely discussed not only in articles on border issues, relations with Georgia, or the negotiation process, but is also raised in publications on identity. Despite Russia’s security guarantees, Georgia is presented as a historical enemy of the South Ossetian people. The historical nature of the confrontation with the mother state and the human losses incurred in this struggle, allow the local authorities to justify the special identity of the South Ossetians and the necessity of independence (Hoch, 2020). Numerous studies on various aspects of identity formation in the countries of Central and Eastern Europe (Elias et al., 1986), the CIS (Vendina et al., 2014) and some post-Soviet unrecognized states (Politika …, 2020) show that “victimization” of one’s own history plays an important role both for the internal and external legitimization of a new polity and its leadership. In South Ossetia, victimization serves as a means of glorifying the current elites and makes it possible to explain the complexity of the socioeconomic situation due to the costs of the recent struggle for independence.

However, in 2010, almost 25% of all publications were devoted to economic topics and related issues of domestic policy. Almost 80% of the materials in this column concerned the post-war reconstruction of the republic: the repair of destroyed and the construction of new housing, the construction and repair of schools and hospitals, etc. The main source of funds for large-scale repair and construction work was the investment program of Russia, with which hopes for an early improvement in life were associated. The discourse widely discussed the specific results of the Investment Program and the opportunities it opened, but the focus was also on the issues of efficient spending of funds and corruption.

Since mid-2010, the focus of public attention has been a possible “loss of sovereignty” due to total economic and political dependence on Russia. The main source of irritation was the “landing” of Russian officials who were sent to the country not only to control the spending of funds and to implement the Investment Program, but also to work in various ministries and departments of the republic. The appointment of Russian citizens to the highest positions and the dominance of the Russian language at the meetings of the Parliament and the Government were considered as elements of colonial administration that threaten national identity. Russian officials were accused of corruption, but most importantly of a lack of understanding of local realities and “real development priorities,” which consisted in the development of local culture.^10^ The tone of the articles changed dramatically only after the meeting of the leadership of the Republic of South Ossetia with the Prime Minister of Russia V. Putin and the conflict quickly subsided.^11^

Another important topic was the discussion of the Development Strategy and its economic, infrastructural, social and institutional goals. The participants of the discussion, represented primarily by local businessmen and officials, saw the development of agriculture, the production of mineral water and tourism among the main priorities. At the same time, tourism, due to its high tourist potential, was characterized as “an exceptionally promising direction,” but requiring very significant public investment.^12^

The strategy, adopted 3 years later, took into account these priorities, which, according to the interviewed experts, remain relevant today. Much more disagreement is caused by the assessment of possible threats that can hinder the achievement of goals. In the Strategy, among such threats is the deterioration of the demographic situation, the moral and physical depreciation of fixed assets, the critical state of infrastructure and the lack of qualified personnel. The strategy does not consider insufficient external legitimacy and a low level of legal recognition as a challenge, and, on the contrary, characterizes the geographical location as favorable and “conducive to the development of interregional ties.”

This part shows the greatest discrepancy between the Strategy and the media discourse, where the “isolation” of the territory of South Ossetia is presented as one of the main problems in the development of the state. Specific manifestations of isolation include difficulties with marketing products to the Russian market, insufficient reliability of the only transport route, that is, the Transcaucasian Highway (called the “road of life”), energy supply problems, limited opportunities for financial transactions, etc. In the Strategy, these problems do not look to be related to each other and possible solutions of varying degrees of feasibility are proposed only for some of them. Thus, to solve the most acute problems of transport isolation, the Alagir-Tskhinvali railway through the Magsky Pass, parallel to the Transcaucasian Highway, an airport and a “network of heliports” are planned. It is with the implementation of these projects, which are not included in the relevant strategic documents of Russia, that the possibilities of developing our own production facilities are associated.

The media discourse is filled with all kinds of projects. At round tables, conferences, and expert meetings on the implementation of the Strategy, projects were presented to create innovative enterprises in the field of information technology, electronics and electrical engineering.^13^ Attempts were even made to implement some of them, which, however, remained unsuccessful.

In 2019, compared to 2010, the media discourse noticeably changed: its significant “economization” was observed. Georgia still appeared in a dehumanized image of an enemy, which, under the conditions of Russian security guarantees, became a source of primarily “soft threats”: illegal cross-border practices, minor border incidents, diseases of people and farm animals. Economic and related domestic political issues accounted for almost 40% of the materials. Decreasing attention was paid to the issues of post-war reconstruction, while increasing attention was paid to the search for ways and directions of economic development. However, fundamentally new ideas practically did not appear during this period. In a single bundle, the discussion of issues of economic development and stateness of the republic continued; however, the discussion no longer bore a conflicting tone. On the contrary, the discourse on economic and political independence was characterized by a type of duality. On the one hand, the Russian Investment Program was seen as the main factor in the modernization and strengthening of the South Ossetian economy and statehood. At the same time, economic self-sufficiency was presented as a key feature that distinguishes a true sovereign state.^14^ On the other hand, in the context of economic and political relations with Russia, the image of South Ossetia was often “desovereigned”: in many news reports in 2019, the republic appeared as a special Russian region. Thus, in forecasts of socio-economic indicators, the Republic of South Ossetia was guided by the neighboring Republic of North Ossetia-Alania and other regions of the North Caucasian Federal District, and the process of delimitation and demarcation of the border with Russia even caused regret to the President of South Ossetia, who hopes that someday “it will become administrative after all.”^15^

## ECONOMIC REALITY IN THE EYES OF LOCAL EXPERTS

By 2020, the adjustment of the entire range of strategic documents of the republic was already on the political agenda, but the difficult epidemiological situation did not allow progress in resolving this issue. This is also the reason that the discussion of the prospects for the socioeconomic development of South Ossetia occupied a central place in our expert interviews. According to experts, the real socioeconomic situation in South Ossetia is in fact much more complex than it is presented in official strategic documents and media discourse.

Most informants noted that the strategic development plans do not fully take into account the small size of the population (officially 50 000 people live in South Ossetia, but according to expert estimates, it is much smaller, 35 000–45 000), which in itself limits opportunities for economic development. In fact, contemporary research on small countries and ministates with sovereignty convincingly proves that the economic viability of such states is extremely limited and is possible only through economic and military-political cooperation with large states (Sharman, 2017), which often takes the form of client-patron relations (Veenendaal, 2017).

The interviews showed that although the staffing problem in the context of high unemployment is not considered by the local authorities as a serious challenge for economic development, business representatives note a shortage of workers. Neighboring Russian regions are too strong a competitor for the local labor market (Novye …, 2012). Higher salaries, various social guarantees, and, in the future, even higher pensions encourage people of working age to leave for work or permanent residence for Russia. The “post-war syndrome” has also become a specific problem for the remaining able-bodied population: not only the heroes of the war of independence, but even very young people still consider wage labor to be unprestigious.

*For local residents, working for a private person is not held in high esteem. The war is over, but people still want to primarily serve in the armed forces, especially in Russia, where the pay is higher. At worst, people are ready to go to work in the police or government agencies. People have forgotten how to work. My labor costs are, oddly enough, higher than in Krasnodar krai of Russia* (October 2020, private enterprise manager of Russian origin).

Local experts also believe that the small capacity of the domestic market remains one of the key obstacles to economic development. High prices and a poor product range are forcing the population to transfer part of their consumption to the territory of Russia. The Russian military and border guards only partly compensate for the drop in demand from the local population, since most of the supplies they need are also supplied directly from Russia. Nevertheless, the military and their families create additional demand for everyday goods, rental housing, etc. However, this solvent demand becomes one of the causes of noticeable distortions in economic development and leads to higher housing and food prices.

Hopes for external demand, which even today is seen as the only driver of economic development in the conditions of low capacity of the domestic consumer market, were not realized either. As Eurasian integration deepened and the EAEU was launched in 2015, the conditions for trade and economic activity with Russia deteriorated significantly (Egorova and Babin, 2015). According to representatives of business and the Chamber of Commerce and Industry of the Republic of South Ossetia, for small and medium-sized businesses, entering the only accessible Russian market has become an increasingly difficult task every year. This was facilitated by strict clearance rules for goods moving across the border (actually the border of the EAEU), the unrecognized status of the Republic of South Ossetia by other participants in the integration association, as well as the unpreparedness of the business community of South Ossetia to apply the norms of the customs code of the EAEU (primarily, certification of goods and execution of declarations for all commodity positions).

*It is practically impossible for an ordinary South Ossetian businessman to enter the Russian market: he has no experience in processing numerous documents and the consignments of goods are very small. The way out could be to increase the amount of duty-free import of products to and from Russia, but this, apparently, requires coordination with other members of the EAEU. A vicious circle!* (Member of the Board of the Chamber of Commerce and Industry of the Republic of South Ossetia).

Unforeseen difficulties often arise when importing products from Russia. Thus, the delivery of high-tech equipment necessary for the diagnosis and treatment of COVID-19 (tomographs, test systems, computers, etc.) in 2020 was hampered by the lack of permits from foreign manufacturers to re-export them from Russia. All this, according to one of the experts, “required complex political decisions taken by the authorities of the two countries in manual mode.”

The issues of financial mutual settlements between counterparties in domestic and foreign economic activity, the development of lending and investment activities remained unresolved. The recognition of South Ossetia by Russia made it possible to partially solve the problem of external and internal settlements. The International Settlement Bank, specially created for these purposes, has offices not only in the capital of South Ossetia, but also in Russia. In 2016–2017, this organization also opened its representative offices on the territory of the then unrecognized Donetsk and Lugansk People’s Republics.^16^ The official recognition of these polities by the South Ossetian authorities allowed the population and enterprises of the people’s republics to gain access to settlement and cash services and conduct business with contractors in Russia. In turn, the South Ossetian elites gained the opportunity to gain additional benefits by acting as intermediaries in the supply of raw materials from Russia to the Donetsk People’s Republic to metallurgical enterprises, gas stations, etc.

However, such schemes did not contribute to attracting investment in the economy of the Republic of South Ossetia and the development of domestic lending. Partially, this gap was filled by Investment Agency LLC, specially created in 2014, which implemented on the territory of the Republic of South Ossetia: (1) projects of concessional lending for a 10-year period at ten percent per annum (“10–10–10” program); (2) a program to compensate for part of the costs incurred for the purchase of Russian-made equipment by the business; (3) a program of free infrastructural development of the territory for the needs of investors. As a result of the agency’s activities, six enterprises were opened, of which only three (the production of Edis mineral water, the Vincenzo restaurant, and a shopping center in the center of Tskhinvali) continue their work. The rest are closed due to problems with the supply of raw materials (the Rastdon meat processing plant) or difficulties in marketing their own products (the Basalt Fiber plant, Gardens of Iryston farm).

A small part of the informants noted that a large problem is the barrier nature of the state border with Georgia (84% of the total length of the state border of South Ossetia), with which relations are hostile and are reduced to a minimum. The last border conflict near the village of Uista in August 2019 was the reason for the closure of the state border. The situation worsened with the introduction of quarantine measures during the coronavirus epidemic in 2020. This became a real challenge for the local Georgian population, which, after the 2008 war, remained involved in the economic life of Georgia almost as much as the South Ossetian population in the life of neighboring Russia. Until 2019, the resale of cheap and high-quality food products and consumer goods was one of the foundations of the cross-border business of many local Georgians, and Leningor restaurants were popular even among Tskhinvali residents. Pensions, allowances, and salaries received from the Georgian authorities have been and remain a significant source of income for local residents.

*Our teachers and doctors, who worked in local institutions until 2008, continue to receive salaries today from both the Georgian and South Ossetian authorities. Georgian salary comes to cards, and now people have lost the opportunity to withdraw this money. We have the same situation with pensions and various benefits. It is difficult when the border is closed* (woman, aged 50, Leningor, October 2020).

**Fig. 3.**
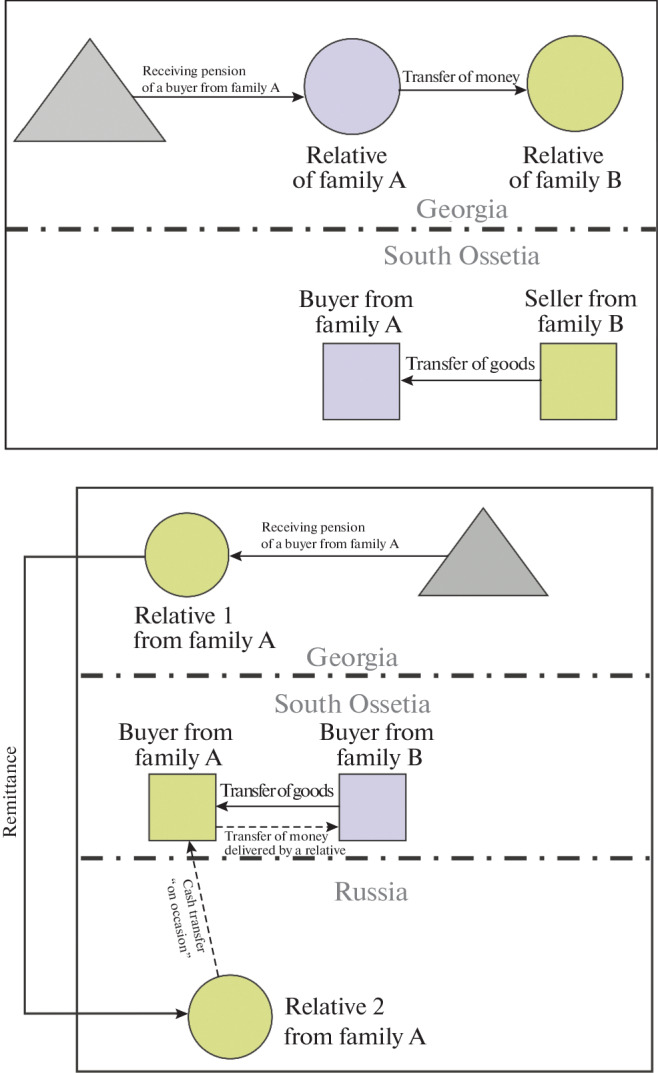
Crossborder payments systems between buyers and sellers of the Leningor district with the participation of relatives on the territory of Georgia and Russia: (a) purchase of consumer goods; (b) purchase of durable goods. *Compiled* by the authors according to the informants.

In an attempt to adapt, local residents came up with their own system of cash and noncash payments, as schematically depicted in Fig. 3. In the first case (see Fig. 3a), the buyer from family A asks his relative in Georgia to transfer money to the relatives of the seller from family B by phone. Upon confirmation of the transfer of funds, the seller from family B transfers the goods to the buyer. This scheme is used to sell consumer goods. In the second case (see Fig. 3b), when the goal is to purchase a more expensive product, a more complex scheme is often used. The buyer from family A asks his relative 1, who lives in Georgia, to make a money transfer to his relative 2, who lives (works or studies) in Russia and visits South Ossetia from time to time. Relative 2 brings money to the buyer, who hands it over to the seller from family B.

However, it is not only the Georgian population that hopes for a softening of the border regime with Georgia. Some South Ossetian experts also want closer ties, but only if the political status quo is maintained.

*The Ergneti market in the late 1990s and early 2000s was one of the largest markets in Transcaucasia. There you could buy everything and sell everything. Many made huge fortunes on this, but many got confused, mired in corruption, like Sanakoev, who betrayed us.*^17^*Such trading is a great opportunity, but it also brings great risks to independence. Now this is not possible for political reasons* (former member of the presidential administration, October 2020).

Lower prices in Georgia for most consumer and industrial goods, easier delivery logistics, and the opportunity to enjoy the benefits of a transit position between Russia and Georgia look like an attractive opportunity to develop the local economy. However, most informants believe that this opportunity should be sacrificed until Georgia recognizes the independence of the republic.

## DISCUSSION OF THE RESULTS AND CONCLUSIONS

According to the concept of Caspersen, gaining viability by unrecognized states is a long process that is full of contradictions between various aspects of internal and external sovereignty. The economy is one of the foundations of the viability of these entities. At the same time, its meaning changes at each stage of the evolution of their statehood. At the first stage (in the case of South Ossetia, 1989–1994), the economy is a key resource for survival and armed struggle against the mother state. At the second stage (1994–2008) it is the material base of state building (institutionalization of the unrecognized state) and post-war reconstruction. At the third stage (after 2008), it is one of the key “sources” of the internal and external legitimacy of the national elites, allowing the “new state” to create and distribute public goods.

Large-scale assistance from Russia allowed the Republic of South Ossetia, despite the complete destruction of the economy during the hot phases of the conflict, to go through all these stages surprisingly quickly. However, the result has been a structurally weak hyperservice economy, with key industries dependent on government demand and investment. These, in turn, are determined by the amount of direct financial support from Russia (subsidies to the budget, investments for restoration, etc.). Therefore, although the Republic of South Ossetia is ahead of its neighbors in terms of per capita income, it is noticeably behind them in terms of GDP per capita and industrial and agricultural production. At the same time, such a high dependence on the patron state does not make South Ossetia an exceptional phenomenon, but rather a typical case among other unrecognized states.

An analysis of the media discourse and official documents of the Republic of South Ossetia showed a stable relationship between the perception of the role of the economy, the degree of securitization of the economic agenda, and the level of statehood development. This connection is manifested in a decrease in interest in the problems of “hard” security, issues of post-war reconstruction, and an increase in attention to the challenges of “soft security,” that is, economic failure and the problems and prospects for socioeconomic development. The assumption was confirmed that in the later stages of the statehood development economic stateness turns out to be the key argument for representing the unrecognized state as a sovereign state. The study showed that this image is formed inconsistently. On the one hand, a developed economy is proclaimed to be one of the key attributes of a “normal” state, on the other hand, the image of South Ossetia is “desovereigned” by its representation as a special Russian region dependent on federal appropriations and focused on domestic Russian socioeconomic goals and development tasks. Interviews with experts also confirmed that, among other arguments in favor of joining Russia, the economic insolvency of the Republic of South Ossetia is the most important one. This argument is shared even by the few supporters of “real” South Ossetian sovereignty.

The media discourse and strategic documents ignore a number of features that limit the possibilities of the socioeconomic development of the republic. Like many other unrecognized states, the Republic of South Ossetia has a small population and a complex transport-geographical and geopolitical position. The effects of a small country here are bizarrely combined with a partially recognized status, limiting opportunities for the development of transport, trade, and financial ties. The COVID-19 pandemic (and the related temporary closure of the borders of the Republic of South Ossetia) demonstrated the vulnerability of the economy of the Republic of South Ossetia, as well as the important role of cross-border practices of the local population (trade, travel, etc.), which, along with direct and indirect assistance from Russia, are an important factor that allow stabilizing the socioeconomic situation in the republic.

The case of South Ossetia confirms our hypothesis that unrecognized (partially recognized) status is not an obstacle in itself, but primarily a restriction of access to markets and a difficulty of financial and trade transactions. The difficulties created by the unrecognized status may well be offset by the patron state and its allies. However, Eurasian integration has become more of a source of problems for South Ossetia, creating obstacles difficult to overcome for local businesses in trade with Russia, which is the only EAEU country that recognizes South Ossetia as a sovereign state.

Some of the experts we interviewed believe that normal relations with Georgia could theoretically help South Ossetia achieve an acceptable level of economic viability by gaining an opportunity to sell its own products and service transit flows to Russia and other countries in the region. However, such a development of events is still perceived with caution not only by politicians, but also by most of the Ossetian society, which sees this as a threat to the sovereignty of the republic.

## Footnotes

1 A detailed critical analysis of such papers is offered by (Broers, 2013; Pegg, 2017; Yemelianova, 2015), etc.

2 In her work, Caspersen refers to the article by Kolstø and Blak-ksirud, who singled out similar phases on the example of the Pridnestrovian Moldavian Republic.

3 https://ugosstat.ru/. Accessed January 21, 2022.

4 Strategy for Socioeconomic Development of the Republic of South Ossetia until 2030, approved by the Decree of the Parliament of the Republic of South Ossetia on August 22, 2013.

5 *Analiz obstanovki v zone gruzino-osetinskogo konflikta. Prognoz razvitiya sobytiy v Yuzhnoy Osetii. 1992* (Analysis of the Situation in the Zone of the Georgian-Ossetian Conflict. Forecast of Development in South Ossetia. 1992), Department of Defense, 1992.

6 Arutyunov, M.G., Linkova, V.V., and Sheinis, V.L., *Dokl. zapiska narodnykh deputatov Rossiyskoy Federatsii Prezidentu Rossiyskoy Federatsii B.N. Yel’tsinu No. 7-16/43-2052 ot 02.09.1992* (Report Note of People’s Deputies of the Russian Federation to the President of the Russian Federation B.N. Yeltsin No. 7-16/43-2052 of September 2, 1992), Department of Defense, 1992.

7 Markin, A., Eduard Kokoity: Commander and President, Politkom.ru, 2008. http://politcom.ru/6729.html.

8 Market of reconciliation, Vestn. Kavkaza, November 28, 2012. https://vestikavkaza.ru/analytics/Rynok-primireniya.html.

9 Markin, A., Eduard Kokoity: Commander and President, Politkom.ru, 2008. http://politcom.ru/6729.html.

10 See for example: God-given right to be yourself, RES, June 28, 2010. https://cominf.org/node/1166483651; Yuri Dzitsoyty: Invited specialists must comply with absolutely all the laws of South Ossetia, RES May 20, 2010. https://cominf.org/node/1166483325.

11 Madina Kozaeva: Russia plays a very important and positive role in the life of the Republic of South Ossetia, RES, June 7, 2010. https://cominf.org/node/11664834722.

12 Ministry of Economic Development of the Republic of South Ossetia: “The front of work is huge, but this is the prospect,” RES, January 8, 2010. https://cominf.org/node/1166482133.

13 See for example: Innovation as an image of the future of South Ossetia, RES, May 11, 2010. https://cominf.org/ node/1166483219; Production of LED lighting systems is planned in South Ossetia, RES, June 5, 2010. https://cominf.org/node/1166483464; Round table “Investment potential of South Ossetia” was held in Tskhinvali, RES, May 6, 2010. https://cominf.org/node/1166483165.

14 Anatoly Bibilov: “2018 was marked for our republic by achievements in various fields,” RES, March 27, 2019. https://cominf.org/node/1166521713.

15 Bibilov highly appreciated the work of the Commission on delimitation and demarcation of the border, RES, May 16, 2019. https://cominf.org/node/1166522651.

16 On February 21, 2022 Russia recognized Donetsk and Lugansk People’s Republics as sovereign states, and in October 2022 both states became part of Russia.

17 D.I. Sanakoev, originally, a South Ossetian politician, Prime Minister of the Republic of South Ossetia in June–December 2001. Since May 2007, the head of a temporary administrative-territorial unit created by order of the President of Georgia M. Saakashvili. According to our informant, the reason for the “betrayal” was blackmail by the Georgian special services about illegal cross-border trade operation.
